# Validation of an automated assay for the measurement of cupric reducing antioxidant capacity in serum of dogs

**DOI:** 10.1186/s12917-016-0760-2

**Published:** 2016-07-02

**Authors:** Camila Peres Rubio, Asta Tvarijonaviciute, Silvia Martinez-Subiela, Josefa Hernández-Ruiz, José Joaquin Cerón

**Affiliations:** Interdisciplinary Laboratory of Clinical Analysis Interlab-UMU, Faculty of Veterinary Medicine, Regional Campus of International Excellence ‘Campus Mare Nostrum’, University of Murcia, Espinardo, Murcia, 30100 Spain; Department of Animal Medicine and Surgery, Veterinary School, University Autonoma of Barcelona, Barcelona, Spain; Department of Plant Biology (Plant Physiology), Faculty of Biology, University of Murcia, Murcia, Spain

**Keywords:** Bathocuproinedisulfonic acid, CUPRAC, Dogs, Oxidative stress, Validation

## Abstract

**Background:**

The objective of the present study was to optimize and validate an automated method to assess the total antioxidant capacity (TAC) in serum of dogs using the cupric reducing antioxidant capacity (CUPRAC) methodology (TAC_c_) with bathocuproinedisulfonic acid disodium salt as chelating agent, evaluating also possible variations due to the use of two different automated analyzers. The method is based on the reduction of Cu^2+^ into Cu^1+^ by the action of the non-enzymatic antioxidants that are present in the sample.

**Results:**

Imprecision was low in both apparatus utilized, and the results were linear across serial Trolox and canine serum samples dilutions. Lipids did not interfere with the assay; however, hemolysis increased the TAC_c_ concentrations. When TAC_c_ concentrations were determined in ten healthy (control) dogs and in twelve dogs with inflammatory bowel disease (IBD), dogs with IBD had lower TAC_c_ concentrations when compared with the healthy dogs.

**Conclusions:**

The method validated in this paper is precise, simple, and fast and can be easily adapted to automated analyzers.

## Background

Oxidative stress is characterized by the inability of endogenous antioxidants to counteract the oxidative damage on biological targets [1, 2]. Antioxidant response can be monitored by analysis of individual biomarkers such as α-tocopherol (vitamin E), carotenoids, glutathione peroxidase, selenium, among others, and/or by assays that measure the total antioxidant capacity (TAC). TAC can be measured by direct methods (e.g., Trolox equivalent antioxidant capacity) [3] that are based on the ability of inhibiting the oxidation of a chemical substance, or by indirect methods based on the determination of the reductive properties of the sample such as the cupric reducing antioxidant capacity (CUPRAC) and the ferric reducing ability of plasma (FRAP) [4, 6, 16]. TAC assays provide a global measurement of the antioxidant capacity of the body, including in some cases possible in vivo interaction between different antioxidants [5, 6].

In humans, a decrease of TAC values has been reported in several conditions, including metabolic syndrome, prediabetes or surgery [7–10]. In dogs, decreased TAC values have been reported after surgery and when anaesthetized with isoflurane [11, 12]. Furthermore, increases in this analyte have been described in demodicosis, parvoviral enteritis, and lymphoma [13–15].

CUPRAC assay evaluates the capacity of the sample in reducing the Cu^2+^ to Cu^1+^ in the presence of a chelating agent. Neocuproine, bathocuproine and bathocuproinedisulfonic acid disodium salt are different chelating agents used for this purpose. This method has been applied to human serum, food, and plant extracts [16–19].

To the authors’ knowledge, no CUPRAC assay has been validated for TAC measurements in canine serum samples. The objective of the present study was to optimize and validate an automated method to assess the TAC in serum of dogs using the CUPRAC methodology (TAC_c_) and the bathocuproinedisulfonic acid disodium salt as the chelating agent. The evaluation was performed using two different automated analyzers; therefore possible variations of the assay due to the use of different equipment were also evaluated.

## Methods

### Chemicals

Trolox (6-hydroxy-2,5,7,8-tetramethylchromane-2-carboxylic acid), potassium chloride (KCl), sodium chloride (NaCl), potassium phosphate (KH_2_PO_4_), bathocuproinedisulfonic acid disodium salt, and copper(II) sulphate (CuSO_4_) were obtained from Sigma-Aldrich. Di-sodium hydrogen phosphate anhydrous (Na_2_HPO_4_) was obtained from Panreac.

### Apparatus

The analyses were performed in the Cobas Mira Plus Biochemical Auto Analyzer (ABX Diagnostic) and in the Olympus AU400 Automatic Chemistry Analyzer (Olympus Europe GmbH).

### Principle of the assay

The CUPRAC assay is based on the reduction of Cu^2+^ into Cu^1+^ by the action of the non-enzymatic antioxidants presented in the sample. The oxidant complex, consisted of Cu^2+^-bathocuproinedisulfonic acid (Cu^2+^-BCS) reacts with the antioxidants of the sample and is reduced to a Cu^1+^-bathocuproinedisulfonic acid (Cu^1+^-BCS), a stable complex which has a maximum absorbance at 490 nm [19]. The antioxidant capacity of the sample is assumed to be equal to the extent of the complex Cu^1+^-BCS formation [19]. The assay used for CUPRAC in the present study was based on the method described by Campos et al. [19] with some modifications.

### Measurement procedure for the Cobas Mira Plus biochemical auto analyzer

In brief, a 5 μL volume of sample was pipetted. Then, 195 μL of the reagent 1 were added and a first read at 500 nm was taken. Subsequently, 50 μL of the reagent 2 were added to the reaction and incubated at 37 °C during 200 seconds. After incubation, a second read at 500 nm was taken and the difference between the first and the second read was used to calculate the antioxidant capacity of the sample. Distilled water was used for blanks.

### Measurement procedure for the Olympus AU400 automatic chemistry analyzer

An amount of 195 μL of reagent 1 and 5 μL of sample were pipetted. A first read at 480 nm was taken before the addition of the second reagent. Then, 50 μL of reagent 2 were added to the mixture and incubated at 37 °C during 280 seconds. A second read at 480 nm was taken and the difference between the first and the second read was used for calculation of the antioxidant capacity of the sample. Distilled water was used instead of sample or standard for blanks.

### Preparation of standards

Trolox solution at a concentration of 2.0, 1.0, 0.5, 0.25, 0.125, 0.0625 and 0.0 mmol/L were used. The results obtained for test samples were compared with a standard curve obtained with Trolox and were expressed as millimoles of Trolox equivalents per liter.

### Optimization of reagents concentrations

To adjust the assay for measurements in canine serum, different concentrations of reagent 1 and reagent 2 were tested with the standards at different concentrations and also with different samples in the Cobas Mira Plus biochemical analyzer.

Reagent 1 was prepared at 0.1, 0.25, 1.0 and 1.6 mmol/L of bathocuproinedisulfonic acid disodium salt in 10 mmol/L phosphate buffer (pH 7.4) while reagent 2 was prepared at 0.1, 0.5, and 0.8 mmol/L of CuSO_4_ in ultrapure water.

The optimal concentrations were selected based on the production of a higher signal with lower background and a lower intra-assay imprecision calculated after analysis of one sample five times in one assay run.

### Analytical validation of the assay

For the analytical validation of the CUPRAC assay, imprecision, accuracy, and sensitivity were evaluated following previously reported protocols [19–22].

#### Imprecision

Imprecision was expressed as coefficient of variation (CV) and was calculated as inter- and intra-assay variations. The CV was calculated as the standard deviation (SD) divided by the mean value (X_mean_) of analyzed replicates x 100 % in the formula CV = (SD x 100 %)/ X_mean_. To determine inter-assay variation, four serum samples were used. Inter-assay CV was determined by analyzing the same samples in separate runs performed on five different days. Five aliquots of each serum sample were stored in plastic vials at −20 °C until analysis. On the day of analysis, the samples were brought to room temperature prior to TAC_c_ measurement. The intra-assay CV was calculated after analysis of four samples five times in one assay run. Intra-assay CV tests were performed for all the different combination of reagents tested, although in the results only appear the values for the final concentration selected for the assay.

#### Accuracy

The accuracy was evaluated through assessment of linearity and spiking recovery. The linearity was evaluated by linearity under dilution, then duplicate determinations of TAC_c_ were made of a canine serum diluted at 1/2, 1/4, 1/8, 1/16 and 1/32 using ultrapure water. Dilution of a Trolox solution (2.0, 1.0, 0.5, 0.25, 0.125, and 0.0625 mmol/L) was also analyzed. For the spiking recovery, two canine serum samples with a known TAC_c_ concentration were mixed in different percentages (12.5, 25, 50, 75 and 87.5 %). The percentages of the measured TAC_c_ concentrations to the expected TAC_c_ concentrations were then calculated.

#### Sensitivity

The detection limit was calculated on the basis of data from 20 replicate TAC_c_ determinations of ultrapure water as mean value plus 3 SDs. The lower limit of quantification (LLOQ) was calculated based on the lowest TAC_c_ concentration that could be measured within a CV less than 15 % [20].

### Effects of hemolysis and lipemia

In order to examine the effect of hemolysis and lipemia, serum samples from three dogs were mixed with various concentrations of hemoglobin and lipids solution, respectively, and TAC_c_ was measured [23]. To study the effects of hemolysis, fresh hemolysate was prepared by the addition of distilled water to packed, washed canine red blood cells from one dog, followed by centrifugation to remove cell debris. The hemoglobin concentration was adjusted to 80, 40, 20, 10, 5, and 0.0 g/L. Ten μL of each concentration were added to three 90 μL samples of canine serum to produce test samples with final hemoglobin concentration of 8, 4, 2, 1, 0.5, and 0.0 g/L, respectively. The 0.0 g/L concentration was reached by adding 10 μL of distilled water to 90 μL of the serum sample. Prepared samples were used to determine the TAC_c_ concentrations.

To investigate the effects of lipemia, a commercial fat emulsion (Lipofundina 20 %; Braun Medical S.S.) with triglycerides concentration of 200 g/L was serially diluted to 50, 25, 12.5, 6.25,3.125 and 0.0 g/L. Ten μL of each dilution were added to 90 μL of the serum samples and were used to determine the TAC_c_ concentration. The final concentrations of triglycerides in the samples were 5, 2.5, 1.25, 0.625, 0.3125, and 0.0 g/L (10 μL of distilled water were added to 90 μL of the serum samples).

The TAC_c_ measurements to evaluate the effect of hemolysis and lipemia in the assay were made in the Olympus AU400.

### Clinical validation

TAC_c_ levels were determined in healthy (control) dogs and dogs with inflammatory bowel disease (IBD). The control samples were from ten (seven males and three females) clinically healthy dogs of several different breeds aged between 3 and 8 years old, that were presented for routine checkups and had normal physical examination. Twelve dogs with IBD were included in this study. They were four female and eight male dogs aged between 3 and 8 years old also of different several breeds. A diagnosis of IBD was made on the basis of clinical signs (vomiting, diarrhea, and weight loss) of at least 3 weeks’ duration, and detection of lymphoplasmacytic inflammation during histologic examination of duodenal biopsy samples following the criteria of Ohta et al. [24]. Exclusion of other causes of chronic gastrointestinal tract signs including urinalysis, abdominal ultrasound, fecal exam, complete blood count and serum biochemistry were made [25, 26].

Blood samples of the healthy and diseased dogs were collected from each via jugular or lateral saphenous venipuncture into tubes without anticoagulant. Samples were centrifuged at 3,500 x g for 5 min at 20 °C. The serum samples were stored in plastic vials at - 20 °C until analysis.

### Statistical analysis

Arithmetic means, medians, intra- and inter-assay CVs were calculated by use of routine descriptive statistical procedures and computer software (Excel 2013, Microsoft; GraphPad Statistics Guide). Linearity under dilution was investigated by linear regression. To compare the TAC_c_ results from both analyzers a Spearman correlation coefficient was used. The influence of hemolysis or lipemia on TAC_c_ concentration was investigated by use of 1-way ANOVA and Dunnett posttests. Interferograms were prepared to show the differences in TAC_c_ concentrations when hemoglobin or lipids were added. Kolmogorov-Smirnov’s test was performed to assess normality of data. Comparison of the TAC_c_ concentrations between healthy dogs and dogs with IBD were made by use of Student’s *t* test once a parametric distribution was given. For all tests, *P* < 0.05 was considered as statistically significant.

## Results

### Analytical validation

**Fig. 1:**
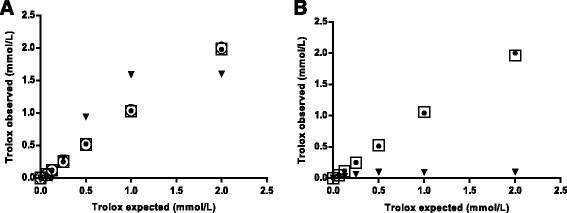
Optimization of both reagent concentrations on a Cobas Mira Plus biochemical analyzer. **a**, Effect of varying concentrations of reagent 1 (▼, 0.1; ○, 0.25; ●, 1.0; □, 1.6 mmol/L) on the calibration curves obtained at a fixed concentration of CuSO_4_ at 0.5 mmol/L. **b**, Effect of varying concentrations of reagent 2 (▼, 0.1; ●, 0.5; □, 0.8 mmol/L) on the calibration curves obtained at a fixed concentration of bathocuproinedisulfonic acid disodium salt at 0.25 mmol/L

**Fig. 2:**
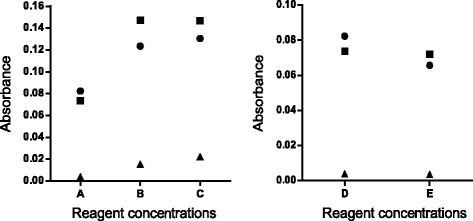
Absorbance obtained of a reagent blank (H_2_O instead of sample) (▲) and of two different canine serum samples (●, ■) studied with different reagent 1 (A, 0.25; B, 1.0; C, 1.6 mmol/L) and reagent 2 concentrations (D, 0.5; E, 0.8 mmol/L). When the reagent 1 was tested, the reagent 2 was fixed at 0.5 mmol/L and when the reagent 2 was tested, the reagent 1 was fixed at 0.25 mmol/L

Results of the optimization of the reagents appear in Figs. 1 and 2. When concentrations of 0.1 mmol/L of reagent number 1 was tested with Trolox, the method lost its linearity at high Trolox concentrations. The other concentrations (0.25, 1.0 and 1.6 mmol/L) showed a similar reaction kinetic, but the background of the reaction increased with increased concentrations. When different concentrations of reagent number 2 were tested with Trolox, the concentration of 0.1 mmol/L did not produce a reaction. Concentrations of 0.5 and 0.8 mmol/L showed a similar reaction kinetic, but when 0.8 mmol/L was used, the background was higher.

When canine serum samples were tested, higher values were obtained when the concentrations of reagents 1 and 2 were increased (Fig. 2). However, the background was also higher. Also, when the reagent 2 was set at 0.8 mmol/L, the imprecision of the assay increased (data not shown). Therefore final concentrations of 0.25 mmol/L bathocuproinedisulfonic acid disodium salt (reagent 1) and 0.5 mmol/L CuSO_4_ (reagent 2) were established as optimal for the assay.

The mean, SD, and the intra- and inter-assay CVs obtained with the CUPRAC assay in the two apparatus are shown in Table 1. Intra-assay CVs were lower than 2 % in all cases. The inter-assay CVs were lower than 7 % and 9 % for the Cobas Mira Plus and Olympus AU400, respectively.

**Table 1:** Mean, standard deviation (SD), intra- and inter-assay CVs variation in TAC_c_ concentrations of canine serum samples (two with lower concentration and two with high concentrations) measured in the two apparatus

Apparatus	Validation parameter	Number of samples	Mean (mmol Trolox equiv./L)	SD	CV (%)
Cobas Mira Plus	Intra-assay	2	0.2325	0.004	1.9
		2	0.3823	0.007	1.8
	Inter-assay	2	0.2254	0.015	6.8
		2	0.3766	0.019	5.1
Olympus AU400	Intra-assay	2	0.2623	0.003	1.2
		2	0.3999	0.003	0.7
	Inter-assay	2	0.2533	0.022	8.6
		2	0.3842	0.030	7.8

*TAC*_*c*_ total antioxidant capacity using the cupric reducing antioxidant capacity methodology and the bathocuproinedisulfonic acid disodium salt, *SD* standard deviation, *CV* coefficient of variation

**Fig. 3:**
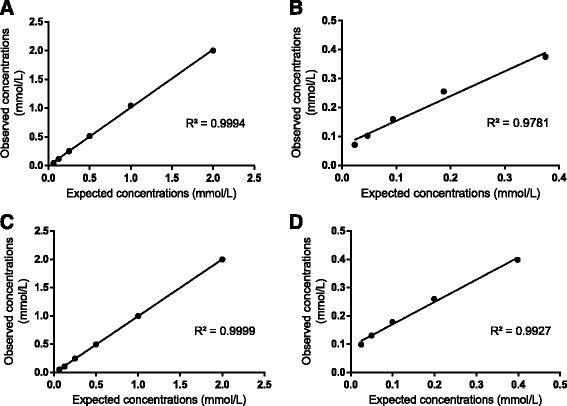
**a** and **b**, Regression lines showing the TAC_c_ concentrations in the Cobas Mira Plus with the Trolox solution and canine serum sample, respectively. **c** and **d**, Regression lines showing the linearity of TAC_c_ concentrations in the Olympus AU400 with the Trolox solution and canine serum sample, respectively. Coefficients of determination (R^2^) are shown

The method showed a high linearity with the Trolox and with canine serum sample in both apparatus (Fig. 3). The results of spiking recovery obtained in the Cobas Mira Plus were between 100 % and 103 % (Table 2), and in the Olympus AU400 were between 97 % and 101 % (Table 3).

**Table 2:** Recovery of CUPRAC assay in canine serum samples evaluated in the Cobas Mira Plus

% sample		Expected (mmol/L Trolox equiv./L)	Detected (mmol/L Trolox equiv./L)	Recovery (%)
High TAC_c_	Low TAC_c_			
100	0	0.3961	0.3961	100
87.5	12.5	0.3753	0.3771	101
75	25	0.3552	0.3581	101
50	50	0.3156	0.3202	102
25	75	0.2760	0.2822	103
0	100	0.2442	0.2442	100

*CUPRAC* cupric reducing antioxidant capacity using bathocuproinedisulfonic acid disodium salt. *TAC*_*c*_ total antioxidant capacity using the cupric reducing antioxidant capacity methodology and the bathocuproinedisulfonic acid disodium salt

**Table 3:** Recovery of CUPRAC assay in canine serum samples evaluated in the Olympus AU400

% sample		Expected (mmol/L Trolox equiv./L)	Detected (mmol/L Trolox equiv./L)	Recovery (%)
High TAC_c_	Low TAC_c_			
100	0	0.2346	0.2346	100
87.5	12.5	0.2571	0.2584	101
75	25	0.2796	0.2785	100
50	50	0.3245	0.3136	97
25	75	0.3695	0.3687	100
0	100	0.4144	0.4144	100

*CUPRAC* cupric reducing antioxidant capacity using bathocuproinedisulfonic acid disodium salt. *TAC*_*c*_ total antioxidant capacity using the cupric reducing antioxidant capacity methodology and the bathocuproinedisulfonic acid disodium salt

The assay detection limit in the Cobas Mira Plus and Olympus AU400 were 0.017 mmol/L (mean ± SD, 0.001 ± 0.005) and 0.003 mmol/L (mean ± SD, 0.0001 ± 0.001), respectively. The lower limit of quantification could not be determined because the CVs were less than 15 % in all cases.

Data of the TAC_c_ obtained with the Cobas Mira Plus were significantly correlated with the data obtained in the Olympus AU400 (r = 0.973, *P* < 0.001).

### Effects of hemolysis and lipemia

**Fig. 4:**
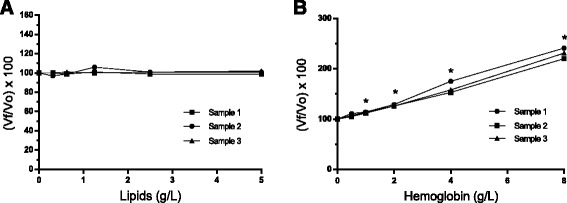
Interferogram for the effect of lipids (**a**) and hemoglobin (**b**) on TAC_c_ concentration. Values for each given concentration (V*f*) are reported as a percentage of the original value (V_o_).* Significantly increased (*P* < 0.05)

Lipids did not interfere with TAC_c_ concentrations (Fig. 4a). The addition of hemoglobin significantly increased the TAC_c_ concentrations, the increase being proportional with the hemoglobin concentration of the sample (Fig. 4b).

### Clinical validation

The TAC_c_ results for serum samples in both apparatus are shown in Table 4. The TAC_c_ concentrations in dogs with IBD were significantly lower than those in healthy dogs when measured with both apparatus.

**Table 4:** Mean ± SD TAC_c_ (mmol/L Trolox equiv./L) concentrations determined in 10 healthy dogs and 12 dogs with IBD

	Healthy dogs	Dogs with IBD	*P* value
Olympus AU400	0.3626 ± 0.02	0.3015 ± 0.07	0.0181
Cobas Mira Plus	0.3904 ± 0.03	0.2936 ± 0.08	0.0049

*TAC*_*c*_ total antioxidant capacity using the cupric reducing antioxidant capacity methodology and the bathocuproinedisulfonic acid disodium salt. *P* < 0.05 was considered significant

## Discussion

In this study, an assay for evaluation of TAC by the CUPRAC method was described and validated for the first time in serum of dogs. This method is automated and uses bathocuproinedisulfonic acid disodium salt. The preparation of the reagents and measurement steps are simpler and faster compared to what has reported in other CUPRAC assays [16], making easier its application on routine analysis. Although in this paper the description of the assay is for automated analyzers, the method can be easily adapted to other formats such as 96 microplate well.

The optimal reagent concentrations determined in this work were different from those described in humans. Campos et al. [19] reported that the final established concentrations were 0.2 and 0.1 mmol/L for reagent number 1 and 2, respectively. Another study reported that the optimal concentrations for the reagent number 1 and 2 were 0.7 and 0.128 mmol/L, respectively [27]. In our study, it was found that higher concentrations of both reagents, especially reagent 2, were needed for the development of an optimal reaction (lower background and higher difference in absorbance between healthy and diseased individuals, and lower imprecision) in dog serum.

The validation results showed that the method has a good precision since all the intra- and inter-assay CVs in both apparatus were lower than 9 %. The method showed a good linearity with dog samples and with serial dilutions of Trolox. Campos et al. [19] also reported a linearity of this assay with Trolox dilutions. The assay showed recovery rates of around 100 % when canine serum samples were mixed at different proportions, indicating that the assay was accurate when measuring the TAC_c_ in canine serum samples.

The validation was performed in two different automated analyzers, the Cobas Mira Plus and Olympus AU400 in order to evaluate their agreement [28] and the repeatability of method in different equipment [29]. In this study, a high correlation between the TAC_c_ results obtained using the Cobas Mira Plus and Olympus AU400 was achieved revealing that this assay can be performed in both apparatus. Regarding the analytical validation results, the two automated equipment also gave similar results with the exception of the detection limit that was higher in the Cobas Mira Plus compared with the Olympus AU400. However, both detection limits were lower than the values usually observed in routine analyses and do not compromise the analytical sensitivity of the assay.

The presence of lipemia and hemolysis can interfere with the results of various analytes leading to an erroneous interpretation if the effect is unknown [23]. In the case of the CUPRAC assay, the lipemia did not interfere with the assay, which is an advantage to use it in clinical setting. However, hemolysis resulted in higher TAC_c_ concentrations. Therefore, results of this assay should be interpreted with caution when hemolytic samples are used.

Dogs with IBD had lower TAC_c_ concentrations than healthy dogs. The IBD is a progressive gastrointestinal tract disorder of unknown cause [25, 30]. During active episodes of IBD, the uncontrolled overproduction of reactive oxygen species could easily overwhelm the antioxidants, which are protective mechanisms, resulting in oxidative damage to cells and tissue. It was suggested that, this event may play a role in the pathogenesis of the disease [31–33]. In humans, the serum TAC, evaluated by the crocin bleaching method, was significantly reduced in IBD patients compared to healthy controls [32]. This is in line with the results of the present study, where our assay was able to demonstrate diminished concentrations of total antioxidants likely in response to oxidative stress in dogs with IBD.

## Conclusions

In conclusion, the automated CUPRAC assay validated in this study can measure the TAC in serum of dogs in a simple, fast and reliable manner and could be adapted to two common automated analyzers. It is expected that this assay could contribute to a wider use of TAC_c_ measurements and therefore the evaluation of the antioxidant status in canine medicine.
